# Deep-sea clawed lobster *Nephropsis stewarti* Wood-Mason, 1872 species complex in the Indo-West Pacific (Crustacea, Decapoda, Nephropidae), with description of a new species

**DOI:** 10.3897/zookeys.1008.59966

**Published:** 2020-12-31

**Authors:** Su-Ching Chang, Tin-Yam Chan, Appukuttannair Biju Kumar

**Affiliations:** 1 Department of Biological Resources, National Chiayi University, Chiayi, 60004, R.O.C., Taiwan National Chiayi University Chiayi Taiwan; 2 Institute of Marine Biology and Center of Excellence for the Oceans, National Taiwan Ocean University, Keelung 20224, R.O.C., Taiwan National Taiwan Ocean University Keelung Taiwan; 3 Department of Aquatic Biology & Fisheries, University of Kerala, Thiruvananthapuram 695581, Kerala, India University of Kerala Kerala India

**Keywords:** Deep-sea, DNA barcoding, lobster, taxonomy

## Abstract

*Nephropsis stewarti* Wood-Mason, 1872 is the most common species of the deep-sea clawed lobster genus *Nephropsis* Wood-Mason, 1872 in the Indo-West Pacific. Morphological comparisons and genetic analyses of extensive material referred to this lobster revealed the presence of three species. The three species differ mainly in body size, development of the intermediate carina on the carapace, position of the lateral pair of rostral teeth, whether the pleonal tergum is granulate, and the spination on the large chelipeds. *Nephropsis stewarti* is restricted to the western central Indian Ocean, and a neotype is selected to fix its identity. The name *Nephropsis grandis* Zarenkov, 2006 is revived with neotype selection for the large form found in the West Pacific and northwestern Australia. The smaller form from southern Taiwan and the Philippines is described as *Nephropsis pygmaea***sp. nov.**

## Introduction

Among the 16 species in the deep-sea clawed lobster genus *Nephropsis* Wood-Mason, 1872 (Nephropidae) (Chan 2010, 2019; Chang and Chan 2020), the type species *N. stewarti* Wood-Mason, 1872 is probably the most well-known in the Indo-West Pacific because of its frequent records showing a wide geographical distribution, large size, and presence at shallower depths compared with other congeners (see for example Miyake 1982; Holthuis 1984, 1991; Baba 1986; Chan and Yu 1988, 1993; Macpherson 1990, 1993; Wadley and Evans 1991; Chan 1997, 1998; Jones and Morgan 2002; Zarenkov 2006; Dineshbabu 2008; Radhakrishnan et al. 2019; Chang and Chan 2019). *Nephropsis stewarti* is a unique species within the genus as it has only one pair of rostral teeth, no distinct spines on the subdorsal carina, a pleon lacking dorsal carina, a telson without erected dorsal spine, and uropodal exopods bearing well-developed diaeresis. Slight differences can be observed among the materials of *N. stewarti* from various localities (see Macpherson 1993), and Zarenkov (2006) considered a large specimen from the Arafura Sea, north of Australia, as a different species, described as *N. grandis* Zarenkov, 2006. Zarenkov’s (2006) specimen (carapace length, including rostrum, 58 mm) is smaller than *N. stewarti* in many other localities (e.g., Macpherson 1990, 1993; Chan 1997; Zarenkov 2006), and the main diagnostic characteristic of *N. grandis* is the spination of the large chelipeds (see Chang and Chan 2019), which is missing in the holotype of *N. stewarti* (Wood-Mason 1873, 1874); therefore, Chan (2010, 2019) tentatively treated *N. grandis* as a junior subjective synonym of *N. stewarti*.

Many reports have illustrated the coloration of specimens identified with *Nephropsis stewarti* (e.g., Miyake 1982; Baba 1986; Chan and Yu 1988, 1993; Wadley and Evans 1991; Jones and Morgan 2002; Chang and Chan 2019). The body of *N. stewarti* was believed to primarily have a whitish color. However, during a recent survey on the decapod crustacean fauna in India carried out by the second author, about 10 *N. stewarti* specimens were observed in a local fishing port, and all were reddish in color. As *N. stewarti* was originally described in the Andaman Sea near India, it was suggested that the currently recognized *N. stewarti* might contain more than one species. This work compared extensive material of *N. stewarti* from various Indo-West Pacific localities, aided by molecular barcoding genetic analysis (Bucklin et al. 2011), and revealed the presence of three species. *Nephropsis stewarti* is restricted to the western central Indian Ocean; a neotype is selected to fix its identity. The name *Nephropsis grandis* can be applied to much of the material from the western Pacific to northwestern Australia; its identity was also fixed by the erection of a neotype. The third, undescribed species, which is smaller in the size compared with the two closely allied species, is present in southern Taiwan and the Philippines; it requires a new name.

## Materials and methods

### Samples

The present study was based mainly on the extensive collection of the *N. stewarti* species complex deposited at National Taiwan Ocean University, Keelung, Taiwan (**NTOU**), supplemented with material from the Muséum national d’Histoire naturelle, Paris, France (**MNHN**); the Department of Aquatic Biology and Fisheries, University of Kerala, India (**DABFUK**); Natural History Museum and Institute, Chiba, Japan (**CBM**); and Senckenberg Museum, Frankfurt am Main, Germany (**SMF**). These materials included topotypic specimens of *N. stewarti* and *N. grandis*. Carapace length (**cl**) was measured dorsally from the orbital margin to the posterior margin of the carapace. The abbreviation (**CP**) preceding the station number indicates the type of the collecting gear (French beam trawl). Morphological terminology generally follows Macpherson (1990) and Holthuis (1991). *Nephropsis stewarti* s.l. is well known in the Indo-West Pacific, having many taxonomic accounts or reports, often with only brief descriptions and without mentioning any of the diagnostic characteristics of the three species identified in this study. Therefore, the synonymy provided is restricted to important taxonomic works related to this species complex.

### Molecular analysis

Although the barcoding gene, cytochrome *c* oxidase I (*COI*), has better resolution for species delimitation (Bucklin et al. 2011), the universal primers (Folmer et al. 1994) failed to amplify the *COI* gene in many western Pacific material of *N. stewarti* (in GenBank there are only two *COI* sequences of *N. stewarti* s.l.: MH428010, 176 bp; LC309102, 713 bp). Therefore, another barcoding gene, 16S rRNA (16S), was used for the present analysis. Twelve of the present specimens from various Indo-West Pacific localities had their 16S newly sequenced. Genomic DNA was extracted from the pleonal somite VI or the pleopod V muscle tissue using a QIAGEN DNeasy Blood and Tissue Kit (QIAGEN). A partial sequence of the 16S rRNA gene was amplified by the primers 16SF (Tsang et al. 2014) and 16SR (Tsang et al. 2009). The PCR amplifications were performed in 25-μL reaction mixtures containing 50–250 ng of the DNA extract, 2.5 μL of 10× polymerase buffer, 3 mM of MgCl_2_, 200 nM of each primer, 200 μM of dNTPs (PROTECH, Taipei, Taiwan), and 1U of ProTaq DNA polymerase (5U/μL, PROTECH). The PCR cycling conditions were as follows: 5 min at 94 °C for initial denaturation; followed by 30 cycles of 30 sec at 95 °C, 40 sec at 47 °C, and 40 sec at 72 °C; and a final extension step for 5 min at 72 °C. The quality of the PCR products was determined by running 5 μL of the reaction on a 1% agarose gel and then sending the sample to a commercial company for further purification (Geneaid) and sequencing (ABI 3730 XL automated sequencer). To examine the accuracy of each sequence, the complementary consensus sequences were aligned by Clustal W, implemented in Bioedit (Hall 1999). The obtained 16S sequences (399–425 bp) were then assembled and aligned by MUSCLE implemented in MEGA v.7 (Kumar et al. 2016), along with the 16S sequences of *Nephropsis* of more than 399 bp, and identified down to the species level in GenBank (Table 1). This revealed that the GenBank sequence EU882882 from a Taiwanese specimen (NTOU M00505) reported by Tshudy et al. (2009) was identical to another GenBank sequence, U96086, of a specimen from Natal, South Africa (Tam and Kornfield 1998). Re-amplification of the 16S gene of the same Taiwanese specimen (NTOU M00505) confirmed that the sequence EU882882 is incorrect (with 7.3% difference). Uncorrected pairwise divergences (*p* distance) among the specimens of *Nephropsis* were performed using MEGA v.7.

**Table 1.** *Nephropsis* Wood-Mason, 1872 material for 16 rRNA sequence analysis. # refers to the same specimen as the sequence EU882882 in GenBank, but with 7.3% divergence. * indicates holotype or neotype.

Species	Locality	Voucher no.	GenBank no.
*N. stewarti*	Andaman Sea	NTOU M02249*	MW301998
	India	DABFUK/AR-ACH-10	MW301999
	Mozambique	MNHN IU-2018-5063	MW302000
	Natal, S. Africa	Unspecified	U96086
*N. grandis*	Indonesia	MNHN IU-2017-9001*	MW302001
	South China Sea	NTOU M02163	MW302002
	the Philippines	NTOU M02251	MW302003
	N. Taiwan	NTOU M00505^#^	MW302004
	S. Taiwan	NTOU M02174	MW302005
*N. pygmaea* sp. nov.	S. Taiwan	NTOU M01898*	MW302006
	S. Taiwan	ZRC2002.0471	AY583891
	the Philippines	NTOU M02263	MW302007
	the Philippines	NTOU M02254	MW302008
*N. serrata*	South China Sea	NTOU M02162	MW302009
	Taiwan	NTOU M00157	EU882881
*N. aculeata*	Massachusetts, USA	Unspecified	U96085
	Unspecified	KC2117	DQ079727
	Mexico	CNCR-21650	EU882884
	Mexico	CNCR-21660	EU882885
*N. rosea*	Mexico	CNCR-21631	EU882886

## Taxonomy

### Family Nephropidae Dana, 1852

**Genus *Nephropsis* Wood-Mason, 1872**

#### Nephropsis stewarti

Taxon classificationAnimaliaDecapodaNephropidae

Wood-Mason, 1872

BEC49E38-830D-5A0A-ACC5-78D200FC396A

Figures 1 , 4A–F

**Figure 1.**
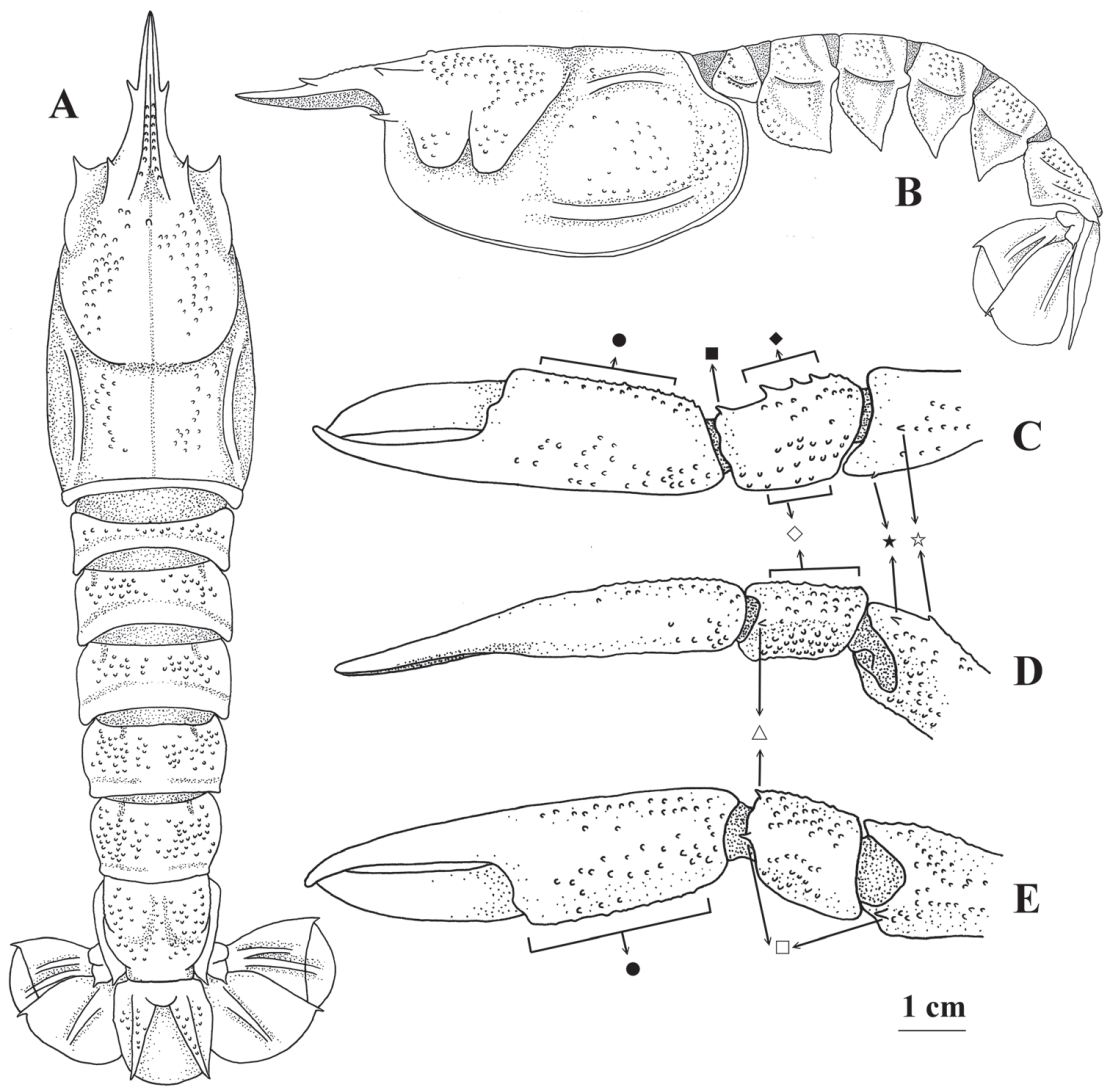
*Nephropsis stewarti* Wood-Mason, 1872, Andaman Sea, RV ‘Dr. Fridtjof Nansen’ stn 135, neotype male cl 46.2 mm (NTOU M02249) **A** carapace and pleon, dorsal **B** same, lateral **C** left pereiopod I, chela to distal part of merus, dorsal **D** same, lateral **E** same, ventral. Pubescence and setae omitted ● Position of spines on inner surface of palm ◇ position of spines on dorsal margin of outer surface of carpus ♦ position of spines on dorsal margin of inner surface of carpus ⏹ dorso-inner distal spine of carpus △ ventro-outer distal spine of carpus ◻ distoventral spine ✰ subdistal dorsal spine of merus ★ position of subdistal outer spine of merus.

**Figure 2.**
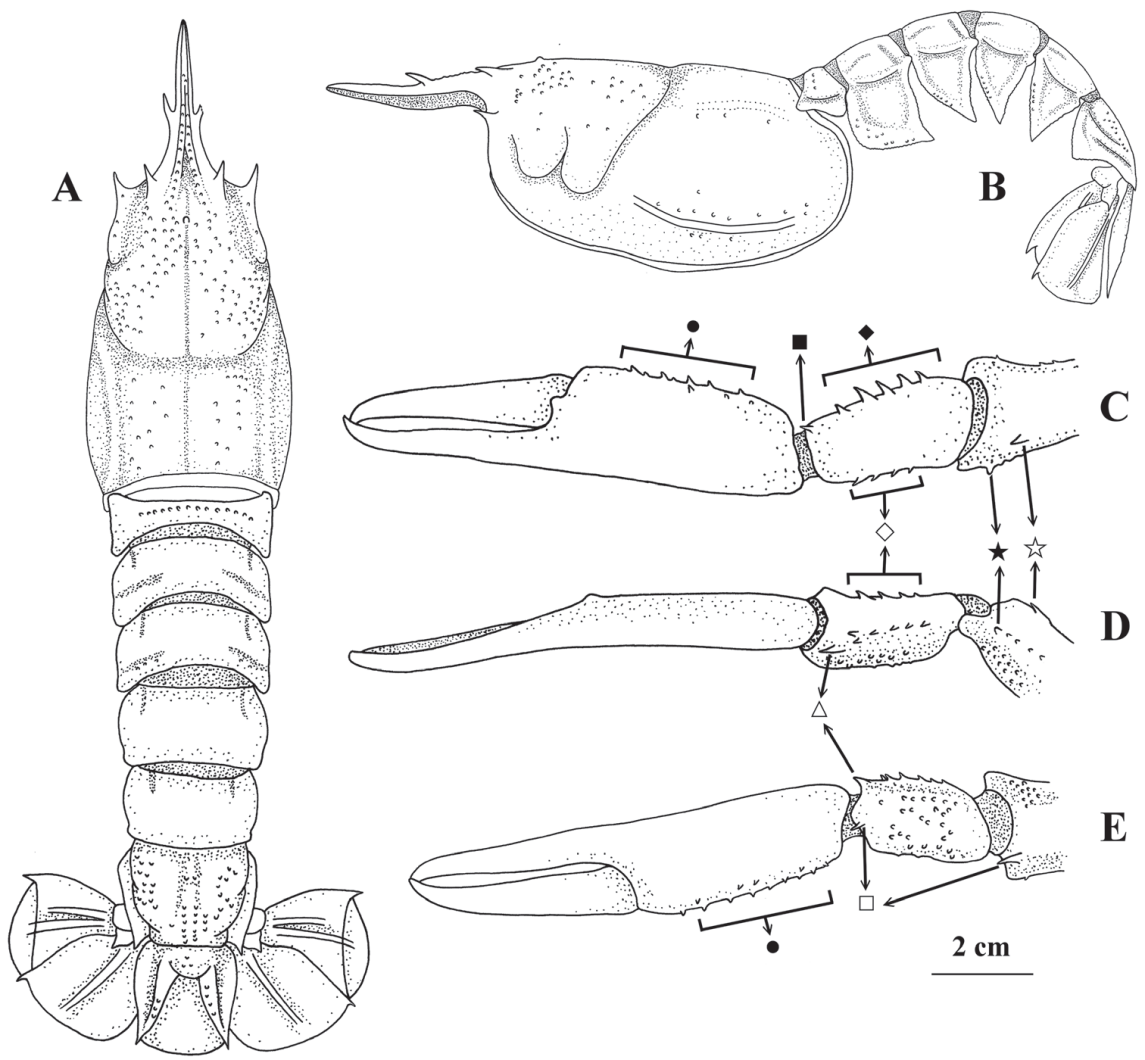
*Nephropsis grandis* Zarenkov, 2006, Tanimbar Islands, Arafura Sea, KARUBAR stn CP59, neotype male cl 64.1 mm (MNHN IU-2017-9001) **A** carapace and pleon, dorsal **B** same, lateral **C** left pereiopod I, chela to distal part of merus, dorsal **D** same, lateral **E** same, ventral. Pubescence and setae omitted ● Position of spines on inner surface of palm ◇ position of spines on dorsal margin of outer surface ♦ position of spines on dorsal margin of inner surface ⏹ dorso-inner distal spine of carpus △ ventro-outer distal spine of carpus ◻ distoventral spine ✰ subdistal dorsal spine of merus ★ position of subdistal outer spine of merus.

Nephropsis Stewarti Wood-Mason 1872: 151 (type locality: Andaman Sea); 1873: 60; 1874: 40, pl. 4-1–3, 5, 7; 1885: 71; Alcock 1894: 230; 1901: 159; Anderson 1897: 96; Alcock and Anderson 1899: 286.
Nephropsis stewartii .–Alcock and Anderson 1894: 161; 1896: pl. 27-figs 1, 1a; Lloyd 1907: 3; Ramadan 1938: 124, text-fig. 1; Thomas 1979: 43.
Not Nephropsis Stewarti.–De Man 1916: 112, pl. 3-fig. 17. [= Nephropsis serrata Macpherson, 1993].
Nephropsis stewarti .–Calman 1925: 21; Barnard 1950: 531; Holthuis 1991: 45 (in part), fig. 80; Macpherson 1990: 312 (in part), figs 5e, 10, 11c, d, 16e; Zarenkov 2006: 93 (in part); Radhakrishnan et al. 2019: 112, fig. 3.22.
Nephropsis ? Nephropsis Stewarti.–Balss 1925: 208.
Not Nephropsis stewarti.–Kubo 1965: 629, unnumbered fig.; Miyake 1982: 77, pl. 26-1; Baba 1986: 153, fig. 103; Chan and Yu 1988: 8, pl. 1A; 1993: 83, unnumbered photo.; Holthuis, 1991: 45 (in part); Wadley and Evans, 1991: 39, unnumbered photo; Macpherson 1993: 63; Chan 1997: 415; Jones and Morgan 2002: 83, unnumbered photo; Davie 2002: 391; Zarenkov 2006: 93 (in part), fig. 19; Chang and Chan 2019: 50 (in part), fig. 7. [= Nephropsis grandis Zarenkov, 2006].
Not Nephropsis stewarti.–Macpherson 1990: 312 (in part). [? = Nephropsis grandis Zarenkov, 2006 and/or Nephropsis pygmaea sp. nov.].
Not Nephropsis stewarti.–Chang and Chan 2019: 50 (in part). [= Nephropsis pygmaea sp. nov.].
Not Nephropsis stewarti.–Chang and Chan 2019: 50 (in part), figs 2C, D. [= Nephropsis serrata Macpherson, 1993].

##### Material examined.

***Neotype***: Andaman Sea • male cl 46.2 mm; RV “Dr. Fridtjof Nansen” stn 135, 12°21.96'N, 96°37.32'E, 514 m, 23 May 2015 (NTOU M02249).

##### Other material.

Andaman Sea • 1 male cl 42.3 mm; RV “Dr. Fridtjof Nansen” stn 68, 14°03.72'N, 94°19.08'E, 457 m, 10 May 2015 (NTOU M02250) • 1 female cl 42.6 mm; commercial trawler, 09°34'65"N, 92°43'21"E, 320 m, 13 Nov. 2017 (DABFUK/AR-ACH-7). Andaman Islands • 1 male cl 38.4 mm, 1 female cl 33.7 mm; A185, commercial trawler, 13 Nov. 2017 (DABFUK/AR-ACH-8). INDIA • 1 male cl 47.0 mm; Sakthikulangara fishing harbor, Kollam district, Kerala, commercial trawler, Nov. 2013 (DABFUK/AR-ACH-9) • 1 male cl 50.8 mm, 2 ovigerous females 42.4 and 48.7 mm; 4 Mar. 2019 (DABFUK/AR-ACH-10) • 2 ovigerous females cl 46.8 and 49.2 mm (DABFUK/AR-ACH-11). Mozambique • 1 male cl 52.7 mm; Mainbaza stn CP3138, 25°12.13'S, 35°21.07'E, 700–707 m, 10 Apr. 2009 (MNHN IU-2018-5063).

##### Diagnosis.

Rostrum bearing one pair of dorsolateral teeth usually situated near mid-length of rostrum. Carapace with subdorsal carinae granulate, without distinct spine or tooth-like process; supraorbital and antennal spines present, lacking post-supraorbital spine; post-cervical groove U-shaped in dorsal view; intermediate and lateral carinae well marked. Large cheliped (pereiopod I) with inner surface of palm lacking distinct spines; carpus with strong distoventral, ventro-outer distal (rarely absent), and dorso-inner distal spines, inner surface with dorsal margin generally bearing 2–4 spines, outer surface without distinct spines; merus bearing subdistal dorsal, subdistal outer and distoventral spines. Pleon finely granulate, without median carina; pleura lacking spine on anterior margins. Telson without erected dorsal median spine near base. Uropodal exopods with complete diaeresis.

##### Description.

Body covered with long or short pubescence, rather thick on anterior two pereiopods, dorsal carapace, and pleonal tergum.

Carapace finely granulated (Fig. 1A, B); rostrum 0.4–0.5× as long as carapace, with 1 pair of teeth usually situated near mid-length of rostrum; subdorsal carinae granulate, without distinct spine or tooth-like process; supraorbital and antennal spines well developed, post-supraorbital spine absent; cervical, postcervical, and hepatic grooves present, with post-cervical groove U-shaped in dorsal view; intermediate and lateral carinae both well marked; gastric tubercle located near supraorbital spine, their distance being approximately 0.4× distance between gastric tubercle and post-cervical groove; distance between orbital margin and post-cervical groove 1.2–1.5× longer than the distance between post-cervical groove and posterior margin of carapace.

Large cheliped (pereiopod I), generally granulate (Fig. 1C–E) but less granular in females; inner surface of palm granular but without distinct spines; chela 3.0–4.0× as long as wide, males generally having relatively longer chela (> 3.5×), whereas females having shorter chela (< 3.5×); carpus with strong distoventral spine, ventro-outer distal spine (rarely absent) and dorso-inner distal spine, inner surface with dorsal margin bearing 2–4 (rarely 1) spines, outer surface without distinct spines; merus bearing subdistal dorsal spine, subdistal outer spine and distoventral spine.

Pereiopod II with carpus 0.5–0.7× palm length. Pereiopod III with carpus 0.4–0.5× as long as palm; merus 1.4–2.1× as long as carpus. Pereiopods IV and V with dactyli 0.4–0.6× as long as propodi.

Entire pleon finely granulate (Fig. 1A, B) without median carina; pleura lacking spine on anterior margins, each terminating ventrally into blunt to sharp spine. Telson without erected dorsal median spine near base. Uropodal exopods with complete diaeresis.

Eggs spherical and 2.2–2.7 mm in diameter.

##### Distribution.

Known with certainty in the western to northeastern Indian Ocean from the eastern coast of South Africa to the Andaman Sea. Found at depths of 250–1520 m and perhaps even 1720 m, but mostly less than 1000 m (see Alcock 1901; Macpherson 1990; Zarenkov 2006).

##### Color in life.

Body varies from whitish to reddish (Fig. 4; Radhakrishnan et al. 2019: fig. 3.22). Eyes and antennal peduncle always whitish. Distal parts of pereiopods II–V, pleopods always reddish. Large cheliped and pleonal tergum, whitish to orange. Pleonal pleura and uropods purple to reddish. Antennal and antennular flagella orange to reddish. Pubescence grayish to reddish. Eggs orange.

##### Remarks.

Although the present Indian specimens have a very reddish color, a comparison with Andaman Sea topotypic specimens and material from Mozambique Channel revealed wide color variations in *N. stewarti*, from whitish to reddish (Fig. 4). Specimens with different colors in the western and northern Indian Ocean are genetically very similar, with 1.0% or less sequence divergence in 16S (Table 2). However, large genetic divergences (16S sequence divergence 3.8–7.3%) exist between the material from the western and northern Indian Ocean, and that from the western Pacific (including the South China Sea and Arafura Sea) and northwestern Australia (Table 2). Such genetic differences are greater than those between *N. rosea* Bate, 1888 and *N. aculeata* Smith, 1881 (3.1–3.7%) and between *N. serrata* Macpherson, 1993 and *N. stewarti* s.l. (lowest 2.8%).

**Table 2.** Uncorrected divergences (*p*-distance) of the 16S gene (399–521 bp) amongst the *Nephropsis stewarti* Wood-Mason, 1872 species complex and *Nephropsis* sequences available in GenBank (excluding those not identified to species and < 399 bp). Number in parentheses refers to number of individuals. Numbers in shade refer to intraspecific divergences. * indicates holotype or neotype.

16S		*N. stewarti*				*N. grandis*					*N. pygmaea* sp. nov.			*N. serrata*		*N. aculeata*		
		Andaman Sea*	India	Mozambique	Natal	Indonesia *	South China Sea	Philippines	N. Taiwan	S. Taiwan	S. Taiwan*	S. Taiwan	Philippines (2)	South China Sea	Taiwan	Massachusetts	unspecified	Mexico (2)
*N. stewarti*	Andaman Sea*																	
	India	0.005																
	Mozambique	0.002	0.007															
	Natal, S. Africa	0.005	0.010	0.002														
*N. grandis*	Indonesia*	0.064	0.064	0.064	0.068													
	South China Sea	0.064	0.064	0.064	0.068	0.009												
	Philippines	0.064	0.064	0.064	0.068	0.009	0.005											
	N. Taiwan	0.069	0.068	0.068	0.073	0.016	0.019	0.019										
	S. Taiwan	0.069	0.068	0.068	0.073	0.016	0.019	0.019	0.000									
*N. pygmaea* sp. nov.	S. Taiwan*	0.040	0.040	0.040	0.044	0.075	0.075	0.075	0.085	0.085								
	S. Taiwan	0.048	0.048	0.048	0.051	0.084	0.084	0.084	0.094	0.094	0.007							
	Philippines (2)	0.038	0.038	0.038	0.041	0.078	0.078	0.078	0.083–0.087	0.083–0.087	0.012	0.019	0.005					
*N. serrata*	South China Sea	0.033	0.028	0.036	0.039	0.078	0.078	0.078	0.088	0.088	0.036	0.043	0.038					
	Taiwan	0.033	0.028	0.030	0.033	0.078	0.078	0.078	0.088	0.088	0.028	0.028	0.030	0.013				
*N. aculeata*	Massachusetts	0.066	0.061	0.066	0.068	0.073	0.073	0.073	0.080	0.080	0.073	0.080	0.071	0.054	0.063			
	unspecified	0.066	0.061	0.066	0.068	0.068	0.068	0.068	0.075	0.075	0.073	0.080	0.071	0.059	0.063	0.015		
	Mexico (2)	0.064	0.059	0.064	0.068	0.066	0.066	0.066	0.073	0.073	0.071	0.079	0.068	0.057	0.063	0.015	0.000	0.000
*N. rosea*	Mexico	0.053	0.047	0.054	0.059	0.064	0.069	0.069	0.071	0.071	0.059	0.067	0.057	0.050	0.053	0.037	0.032	0.031

**Figure 3.**
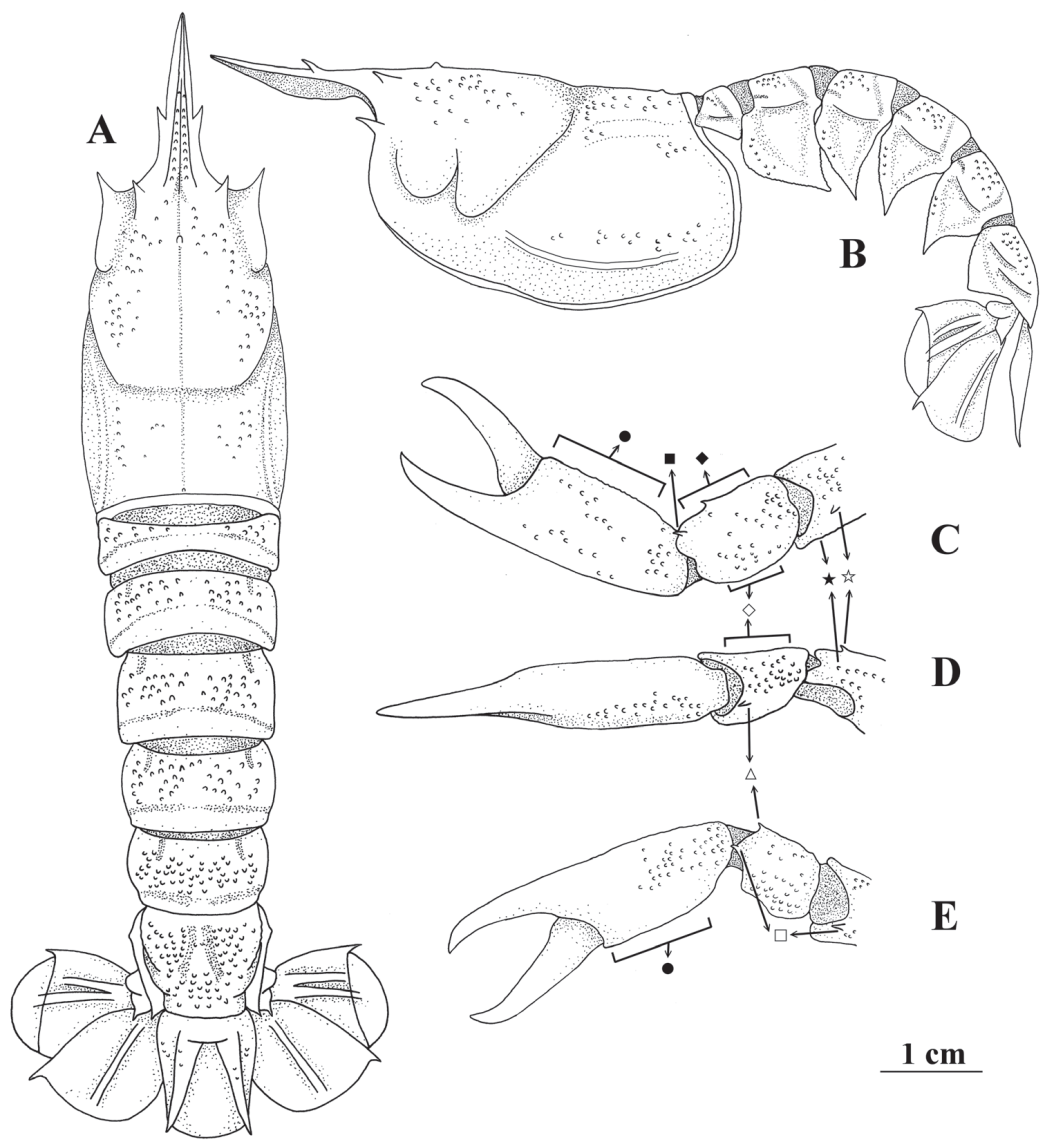
*Nephropsis pygmaea* sp. nov., Donggang, Pingtung County, Taiwan, holotype male cl 25.6 mm (NTOU M01898) **A** carapace and pleon, dorsal **B** same, lateral **C** left pereiopod I, chela to distal part of merus, dorsal **D** same, lateral **E** same, ventral, pubescence and setae omitted ● position of spines on inner surface of palm ◇ position of spines on dorsal margin of outer surface ♦ position of spines on dorsal margin of inner surface ⏹ dorso-inner distal spine of carpus △ ventro-outer distal spine of carpus ◻ distoventral spine ✰ subdistal dorsal spine of merus, ★ position of subdistal outer spine of merus.

*Nephropsis grandis*, previously considered to be a synonym of *N. stewarti*, has a type locality in the Arafura Sea (Zarenkov 2006). Of the three characteristics proposed by Zarenkov (2006: table 1) to separate *N. grandis* from *N. stewarti*, the shape of the distal part of the rostrum has been shown to be variable. The subdistal outer spine on the merus of the large cheliped is present in all of the western and northern Indian Ocean specimens (Fig. 1C, D) but can be present or absent in the western Pacific and northwestern Australia material (Figs 2C, D, 3C, D). The degree of development of the lateral carina on the carapace is similar in all the Indo-West Pacific material (Figs 1B, 2B, 3B). However, the intermediate carina on the carapace is well developed in all of the western and northern Indian Ocean material (Fig. 1A, B) but is indistinct in the western Pacific and northwestern Australia specimens (Figs 2A, B, 3A, B). Moreover, a pair of lateral rostral teeth is usually situated around the mid-length of the rostrum in the western central Indian Ocean material (with only one exception; a cl 42.3 mm ovigerous female of DABFUK/AR-ACH-10) but mostly in a position distinctly posterior to the middle of the rostrum in the specimens from the western Pacific and northwestern Australia (except in three specimens; one in *N. grandis*: NTOU M02177, and two in *N. pygmaea* sp. nov.: NTOU M02168, NTOU M02262).

**Figure 4.**
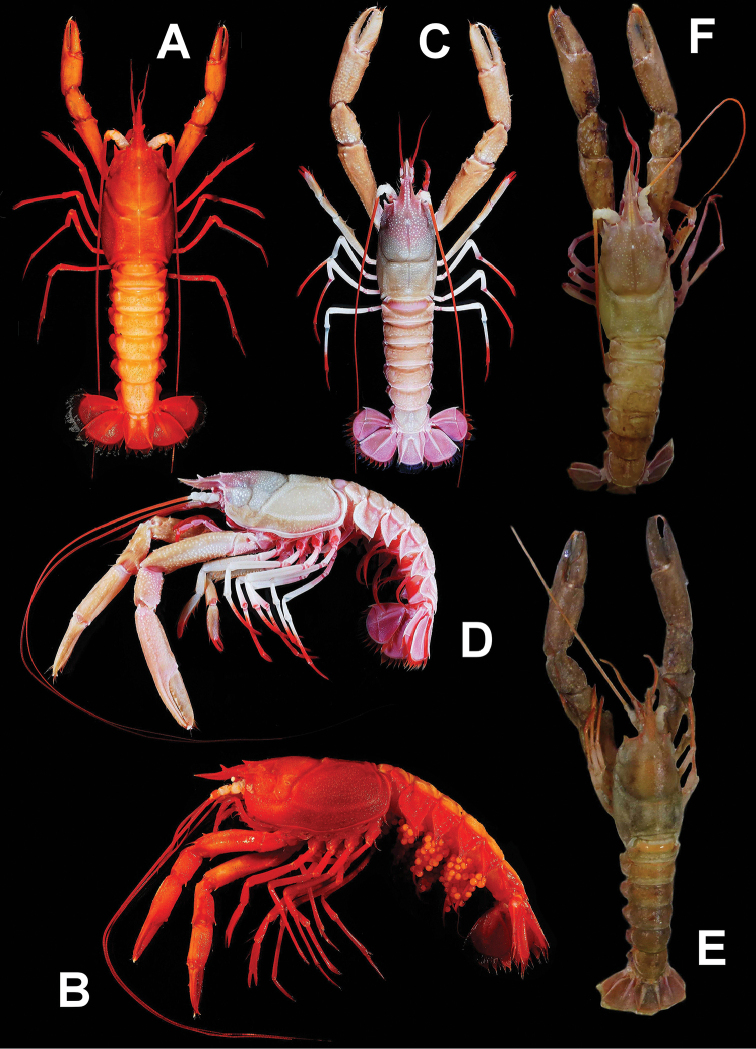
**A–F***Nephropsis stewarti* Wood-Mason, 1872 **A, B** Sakthikulangara fishing harbor, India, ovigerous female cl 42.4 mm (DABFUK/AR-ACH-10) **C, D** Mozambique, MAINBAZA stn CP3138, male cl 52.7 mm (MNHN IU-2018-5063) **E** Andaman Sea, RV ‘Dr. Fridtjof Nansen’ stn 135, neotype male cl 46.2 mm (NTOU M02249) **F** Andaman Sea, RV ‘Dr. Fridtjof Nansen’ stn 68, male cl 42.3 mm (NTOU M02250) **A, C, E, F** dorsal habitus **B, D** lateral habitus **E, F** photographed by PN Psomadakis.

*Nephropsis stewarti* was originally described from a single female specimen lacking large chelipeds, and collected from Ross Island of the Andaman Islands (Wood-Mason 1873, 1874). Soon after its discovery, many more specimens of this species were collected in India (see Alcock 1901); however, the holotype (supposed to have registration number 1404) is no longer extant, although having been held by the Zoological Survey of India, Calcutta (S Mitra, Zoological Survey of India, Calcutta, personal communication). As the *N. stewarti* species complex has now been found to contain at least three species, in order to fix the identity of *N. stewarti*, a recently collected Andaman Sea specimen (NTOU M02249) with color (Fig. 4E) and genetic information (Table 1) and that is very close to the type locality, is herein selected as the neotype of this species. The neotype fits well with the description of the holotype (Wood-Mason 1873, 1874), particularly in terms of the eye being rudimentary, bearing one pair of lateral rostral teeth, exopod of uropod with distinct diaeresis, and generally being similar to the figures provided for the holotype (Fig. 1A, B; Wood-Mason 1874: pl. 4-1–3).

Because material from northwestern Australia in the eastern Indian Ocean is now considered to belong to *N. grandis* instead of *N. stewarti*, re-examination of the specimens from northeastern Sumatra in the eastern Indian Ocean, reported by Balss (1925), will be necessary to determine if they represent *N. stewarti*, despite being collected near the type locality of the latter species. Re-examination of the “*N. stewarti*” material, reported by Chang and Chan (2019), revealed that all but one female (NTOU M02162) from the South China Sea belong to either *N. grandis* or the new species described below. This particular female specimen has the subdorsal carina on the carapace bearing small spines; therefore, it actually represents *N. serrata* (also see Tables 1, 2).

Although a red or white body color is thought to be specific for *Nephropsis* (see Chang and Chan 2019), this is not the case for *N. stewarti*. The Indian material appears to be much redder (Fig. 4A, B; Wood-Mason 1885; Alcock and Anderson 1899; Radhakrishnan et al. 2019: fig. 3.22) and has more distinct granules on the pleon, sometimes even arranged like a median carina. Thomas (1979), however, mentioned that his *N. stewarti* material from the Gulf of Mannar had a greenish-yellow color and deep red appendages. Color photographs are available for two of the Andaman Sea specimens examined (NTOU M02249, M02250). One (Fig. 4F) has a rather white body like the Mozambique specimen (MNHN IU-2018-5063, Fig. 4C, D), except for the pereiopods II to V, which are entirely pale purple. The neotype (Fig. 4E) has a body that is generally pale orange (which is intermediate between red and white). Color information on more specimens from different areas of the central western Indian Ocean will be necessary to understand whether material from the same locality also exhibits large variations in body color for this species.

#### Nephropsis grandis

Taxon classificationAnimaliaDecapodaNephropidae

Zarenkov, 2006

4468A3DC-274F-550D-B8DE-A985A6FB9198

Figures 2 , 5A, B

Nephropsis grandis Zarenkov 2006: 86, figs 5–7 (type locality: Arafura Sea).
Nephropsis stewarti .–Kubo 1965: 629, unnumbered fig.; Miyake 1982: 77, pl. 26-1; Baba 1986: 153, fig. 103; Chan and Yu 1988: 8, pl. 1A; 1993: 83, unnumbered photo; Holthuis 1991: 45 (in part); Wadley and Evans 1991: 39, unnumbered photo; Macpherson 1993: 63; Chan 1997: 415; Jones and Morgan 2002: 83, unnumbered photo; Davie 2002: 391; Zarenkov 2006: 93 (in part), fig. 19; Chang and Chan 2019: 50 (in part), fig. 7. [not Wood-Mason 1872].
? Nephropsis stewarti.–Macpherson 1990: 312 (in part). [not Wood-Mason 1872].

##### Material examined.

***Neotype***: Indonesia • male cl 64.1 mm; Tanimbar Islands, Arafura Sea, Karubar stn CP59, 08°20'S, 132°11'E, 405–399 m, 31 Oct 1991 (MNHN IU-2017-9001).

##### Other material.

Japan • 1 male cl 43.5 mm; Suruga Bay, off Numazu, commercial trawler, 34°44.37'N, 138°41.13'E, 350 m, 20 Apr. 2016 (CBM-ZC 14212) • 1 male cl 46.0 mm, 1 female cl 36.0 mm; Tosa Bay, off Mimase, 16 Jan.–14 Feb. 1963 (SMF 18328) • 2 males CL 32.0, 41.0 mm, 1 female cl 27.5 mm; 1961–1963 (SMF 24678). TAIWAN • 1 female cl 44.8 mm; Dasi fishing port, Yilan County, commercial trawlers, 10 Sept. 1984 (NTOU M02165) • 1 male cl 45.3 mm, 2 females cl 39.7 and 39.8 mm; Sept. 1992 (NTOU M02171) • 1 male cl 38.9 mm; Aug. 2003 (NTOU M00505) • 1 male cl 41.9 mm; 29 May 2008 (NTOU M02177) • 1 female cl 32.4 mm; 12 Apr. 2012 (NTOU M02178) • 1 male cl 19.8 mm; 14 Aug. 2013 (NTOU M02179) • 1 male (carapace damaged), 1 female cl 40.7 mm; Nanfang-ao fishing port, Yilan County, commercial trawlers, 2 May 1985 (NTOU M02166) • 1 male cl 28.2 mm, 1 female cl 40.5 mm; 20 Apr. 1988 (NTOU M02167) • 1 male cl 31.8 mm; 12 Nov. 2004 (NTOU M02176) • 2 males cl 29.0 and 32.7 mm; Donggang fishing port, Pingtung County, commercial trawlers, 27 Dec. 1997 (NTOU M02174) • 1 male cl 31.1 mm, 2 females cl 30.2 and 40.6 mm; locality not specified, 1993 (NTOU M02172). South China Sea • 1 female cl 45.9 mm; Dongsha (Pratas), Jun. 1991 (NTOU M02170) • 1 male cl 15.9 mm; Zhongsha 2015 stn CP4137, 19°53.059'N, 114°21.678'E, 536–524 m, 23 Jul. 2015 (NTOU M02161) • 1 male cl 12.8 mm; stn CP4155, 16°13.60'N, 115°01.61'E, 526–510 m, 28 Jul. 2015 (NTOU M02163). Philippines • 1 female cl 32.0 mm; PANGLAO 2005 stn CP2384, 8°46.2'N, 123°16.1'E, 647–613 m, 29 May. 2005 (NTOU M02251). Indonesia • 1 female cl 51.1 mm; Tanimbar Islands, Arafura Sea, Karubar stn CP39, 07°47'S, 132°26'E, 477–466 m, 28 Oct. 1991 (MNHN IU-2017-9002) • 1 female cl 25.4 mm; stn CP59, 08°20'S, 132°11'E, 405–399 m, 31 Oct. 1991 (MNHN IU-2018-5062). North West Australia • 1 male cl 53.7 mm; 18°19'S, 117°149'E, 25 Feb. 1986 (NTOU M02252).

##### Diagnosis.

Rostrum armed with a single pair of lateral teeth usually situated posterior to mid-length of rostrum. Carapace with subdorsal carinae granulate, lacking distinct spine; supraorbital and antennal spines present; post-supraorbital spine absent; postcervical groove U-shaped in dorsal view; intermediate carina weak, indistinct. Large cheliped (pereiopod I) with inner surface of palm usually armed with row of distinct spines; carpus with strong distoventral, ventro-outer distal, and dorso-inner distal spines, inner surface usually with 2–4 spines along dorsal margin and several small spines on ventral margin, both dorsal and ventral margins of outer surface spinose; merus with subdistal dorsal and anteroventral spine, generally also bearing a spine or sharp tubercle on subdistal outer surface. Pleon generally smooth and lacking mid-dorsal carina; pleura each with unarmed anterior margin. Telson without erected mid-dorsal spine near base. Uropodal exopods with complete diaeresis.

##### Description.

Body covered with long or short pubescence, rather thick on dorsal carapace, pleonal tergites and anterior two pereiopods. Carapace finely granulated (Fig. 2A, B); rostrum 0.4–0.8× as long as carapace (proportionally longer in small individuals), bearing 1 pair of lateral teeth usually situated posterior to mid-length of rostrum; subdorsal carinae granulate, lacking distinct spine; strong supraorbital and antennal spines present; post-supraorbital spine absent; cervical, postcervical, and hepatic groove well-marked, with post-cervical groove U-shaped in dorsal view; intermediate carina weak, indistinct; lateral carina distinct; distance between gastric tubercle and supraorbital spines 0.3–0.4× distance between gastric tubercle and postcervical groove; distance between orbital margin and postcervical groove 1.3–1.5 (rarely 1.6)× distance between post-cervical groove and posterior margin of carapace.

Large cheliped (pereiopod I), generally with smooth surface (Fig. 2C–E); chela 2.7–4.7 (mostly 2.9–3.8)× as long as wide; inner surface of palm generally armed with row of distinct spines except for very small individuals; carpus with strong distoventral spine, ventro-outer distal spine, and dorso-inner distal spine, inner surface having 2–4 spines (sometimes only one in very small individuals) along dorsal margin and several small spines on ventral margin, both dorsal and ventral margins of outer surface spinose (with fewer spines in small young specimens); merus with subdistal dorsal spine and distoventral spine, usually also bearing subdistal outer spine or sharp tubercle. Pereiopod II with carpus 0.5–0.7 (rarely 1)× palm length. Pereiopod III with carpus 0.4–0.5× as long as palm; merus 1.8–2.1× longer than carpus. Pereiopods IV and V with dactyli 0.3–0.6× as long as propodi.

Pleon generally smooth (Fig. 2A, B), lacking median carina, only tergites I and VI granulate; pleura each with unarmed anterior margin and each terminating ventrally into a blunt or sharp spine. Telson without erected dorsal median spine near base. Uropodal exopods with complete diaeresis.

Eggs spherical and approximately 3 mm in diameter (Chan and Yu 1988).

##### Distribution.

Western Pacific and northwestern Australia, known with certainty from Japan, Taiwan, South China Sea, the Philippines, Indonesia (Kai and Tanimbar Islands), Arafura Sea, and northern Australia (Queensland to NW Shelf); at depths of 312–647 m (Macpherson 1993; present study) and perhaps 170–821 m (see Remarks).

##### Color in life.

**Figure 5.**
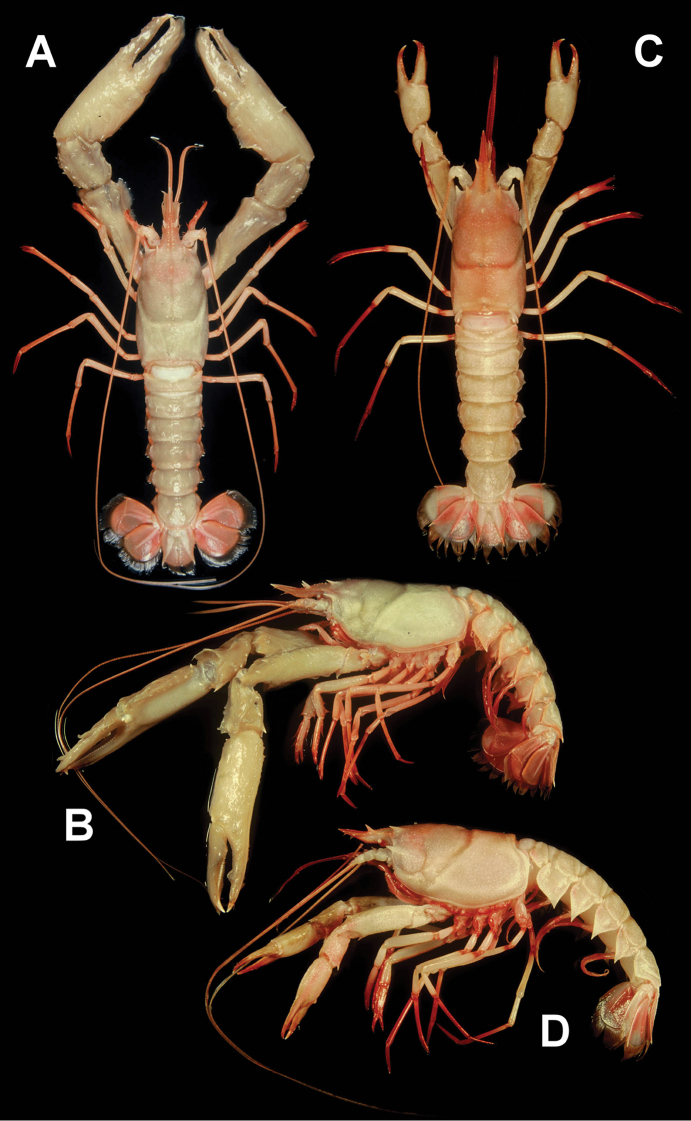
**A, B***N. grandis* Zarenkov, 2006, Dasi fishing port, Taiwan, male cl 45.3 mm (NTOU M02171) **C, D***N. pygmaea* sp. nov., Donggang fishing port, Taiwan, paratype female cl 24.4 mm (NTOU M02168) **A, C** dorsal habitus **B, D** lateral habitus.

Body, including eyes, generally whitish, pubescence grayish to grayish brown (Fig. 5A, B; Miyake 1982: pl. 26–1; Baba 1986: fig. 103; Chan and Yu 1988: pl. 1A; 1993: unnumbered photo; Wadley and Evans 1991: unnumbered photo; Jones and Morgan 2002: unnumbered photo). Rostrum and antennal flagella orange to reddish, sometimes anterodorsal carapace also orange. Antennular flagella whitish to reddish. Maxilliped III and pereiopods II to V white to orange-pink and with distal parts reddish. Large cheliped whitish to somewhat orange. Pleopods and margins of pleonal pleura whitish or reddish. Uropods and distal part of telson pinkish red to reddish. Eggs whitish (Chan and Yu 1988).

##### Remarks.

Although the western Pacific and northwestern Australia material has been shown to be not the true *N. stewarti*, molecular genetic analysis suggests that there are two distinct species (16S sequence divergence as high as 7.5–9.4%, even higher than 3.1–6.3% among *N. serrata*, *N. aculeata*, and *N. rosea*; Table 2). Careful comparisons revealed that these two genetic forms differ, detailed as follows. (1) The body size is large in one form (up to cl 64.1 mm, MNHN IU-2017-9001) but much smaller in the other (up to cl 28.0 mm NTOU M02259). (2) Although the pleon is concealed by thick pubescence, the surfaces of tergites II to V is rather smooth in the large size form (Fig. 2A, B) but distinctly granular in the smaller form (Fig. 3A, B). (3) The large cheliped is also concealed by long pubescence but is more spiny in the large form. In the large form, the carpus is heavily spinose on the outer surface and has 2–4 spines along the dorsal margin of the inner surface (Fig. 2C–E). The inner surface of the palm is also spiny (Fig. 2C, E), except in small young specimens (cl < 20 mm), and the merus generally has a subdistal outer spine or sharp tubercle (Fig. 2C, D) except in one female (MNHN IU-2018-5062). For the large cheliped of the smaller form, the carpus has an outer surface without distinct spines and the inner surface bears only one (mostly) or two spines on the dorsal margin (Fig. 3C–E). The inner surface of the palm lacks distinct spines (Fig. 3C, E), and there is no subdistal outer spine or sharp tubercle on the merus (Fig. 3C, D).

*Nephropsis grandis* was described from a single male collected near the Tanimbar Islands in the Arafura Sea (09°07.5'S, 131°14.9'E, Zarenkov 2006). The holotype is a large specimen (carapace length, including rostrum, 58 mm) and its pleonal tergites I–VI are without granules on the surfaces. The large cheliped in the holotype appears to be rather spinous; however, it is difficult to comprehend the exact spination on the various parts from the original description and illustrations. For example, the holotype appears to lack a subdistal outer spine at the merus of the large cheliped (Zarenkov 2006: table 1: subdistal spine on outer surface of merus of cheliped). Nonetheless, the larger form discussed above generally fits the characteristics described for *N. grandis*, and at present, only the larger form is found in the Arafura Sea. Currently, the holotype of *N. grandis* (ZMMU Ma 5157) cannot be located in the Zoological Museum of Moscow University (V Spiridonov, personal communication). As there are now three species in the *N. stewarti* species complex and each has only subtle differences, it is desirable to fix the identity of *N. grandis*. The current largest specimen (MNHN IU-2017-9001) from the Tanimbar Islands, and with genetic data available, was selected as the neotype of *N. grandis*, thus affixing this name on the larger form in the West Pacific and northwestern Australia. The neotype was collected from a locality very close to that of the holotype (both in the Arafura Sea) and is generally similar to the original figures provided for the holotype (Fig. 2A–C; Zarenkov 2006: figs 5, 6).

*Nephropsis grandis* is genetically distinct from *N. stewarti*, with 6.4–7.3% 16S sequence divergence (Table 2). Other than the differences described under the “Remarks” of *N. stewarti*, these two species also differ in the pleonal tergites II–V, being smooth in *N. grandis* (Fig. 2A, B) but granular in *N. stewarti* (Fig. 1A, B). In *Nephropsis grandis* the outer surface of the large cheliped carpus is spinose (Fig. 2C–E) but this is only granular and without distinct spines in *N. stewarti* (Fig. 1C–E). In large specimens, the inner surface of palm of the large cheliped is also spinose in *N. grandis* (Fig. 2C, E) but still lacks spines in *N. stewarti* (Fig. 1C, E). Macpherson (1993) suggested that the shape of the large chela might be different among the species in the *N. stewarti* species complex. However, this is not supported by the present work, even though the large chelae have sexual dimorphism only in *N. stewarti*.

*Nephropsis grandis* is widely distributed from Japan to Australia. Photographs of the Japanese specimen identified as “*N. stewarti*” from Suruga Bay (CBM-ZC 14212) and with a very short 16S sequence for eDNA metabarcoding (LC430805, 163 bp; Komai et al. 2019) is now confirmed to represent *N. grandis*. The short 16S sequence of this specimen is also identical to the sequence of the present Taiwanese specimens (NTOU M02174, NTOU M00505) assigned to *N. grandis*. The Japanese specimens (SMF 18328), referred to “*N. stewarti*” by Zarenkov (2006), are rather small (two males and one female, cl, including rostrum, 32–41mm) but still have a distinct spine on the outer surface of the carpus of the pereiopod I (Zarenkov 2006: fig. 19A); therefore, they likely represent *N. grandis* instead. The SMF 18328 lot, however, consists of two moderately large specimens (cl 36.0 mm and 46.0 mm). One more lot of “*N. stewarti*” from Japan is held in the Senckenberg Museum (SMF 24678), and there are three specimens within the lot. Although the number and sex of the specimens in the SMF 24678 lot match those reported for SMF 18328 in Zarenkov (2006), their sizes (cl 27.5–41.0 mm) do not match. Nevertheless, photographs of all five of these Japanese specimens in the Senckenberg Museum clearly show that they are all *N. grandis* because of the weak intermediate carina, large cheliped with distinct spines on the outer surface of the carpus, and the merus having subdistal outer spine. For the published photographs of Japanese “*N. stewarti*”, the one of Baba (1986: fig. 103) clearly shows the large cheliped with the inner surface of the palm and the outer surface of the carpus bearing distinct spines. The other photograph of Miyake (1982: pl. 26-1) also shows the large cheliped with the outer surface of the carpus armed with distinct spines, although the spination on the inner surface of the palm is unclear because of the covering of thick pubescence. Thus, it appears that only *N. grandis* is distributed in Japan among the *N. stewarti* species complex. Among the two sequenced Taiwanese specimens of this species, specimen NTOU M00505 was used in a clawed lobster phylogenetic study (Tshudy et al. 2009) as “*N. stewarti*” with GenBank no. EU882882, which has a sequence identical to U96086 from a specimen of *N. stewarti* in Natal, South Africa (Tam and Kornfield 1988). However, re-amplification of the 16S gene of the NTOU M00505 specimen (GenBank no. M302004) revealed that its sequence does not match EU882882 and belongs to the clade of *N. grandis* instead.

The present work revealed that among the *N. stewarti* species complex, both *N. grandis* and *N. pygmaea* sp. nov. are distributed in southern Taiwan and the Philippines, and the true *N. stewarti* is restricted to the Indian Ocean. Re-examination of the Philippines material (with a depth range of 170–821 m) reported as “*N. stewarti*” in Macpherson (1990) is necessary to determine which of these two species they belong to, and whether *N. grandis* can be found in waters as shallow as 170 m and/or as deep as 821 m.

#### Nephropsis pygmaea sp. nov.

Taxon classificationAnimaliaDecapodaNephropidae

C0667053-2621-58CF-A55C-C60C9327F5EB

http://zoobank.org/286FA460-CA9A-465F-B793-22F9F603D4BA

Figures 3 , 5C, D

Nephropsis stewarti .–Holthuis 1991: 45 (in part); Chang and Chan 2019: 50 (in part). [not Wood-Mason 1872].
? Nephropsis stewarti.–Macpherson 1990: 312 (in part). [not Wood-Mason 1872]

##### Material examined.

***Holotype***: Taiwan • male cl 25.6 mm; Donggang, Pingtung County, commercial trawler, 22°11.880'N, 120°22.213'E, 630 m, 2 Oct. 2014 (NTOU M01898).

***Paratypes***: Taiwan • 1 male cl 23.4 mm; Donggang fishing port, Pingtung County, commercial trawler, Jul. 1975 (NTOU M02164) • 1 male cl 21.1 mm, 6 females cl 19.5–26.2 mm; 3 May. 1991 (NTOU M02168) • 2 males cl 19.2 and 21.7 mm; 14 May. 1991 (NTOU M02169) • 1 male cl 22.4 mm; 4 Jun. 1995 (NTOU M02173) • 1 male cl 21.8 mm; 27 Dec. 1997 (NTOU M02175).

##### Other material.

Philippines • 4 males cl 12.5–18.1 mm, 1 female cl 19.1 mm; PANGLAO 2005 stn CP2333, 09°38.2'N, 123°43.5'E, 596–565.5 m, 22 May 2005 (NTOU M02253) • 1 male cl 16.2 mm; stn CP2335, 09°34.3'N, 123°37.8'E, 733–743 m, 22 May 2005 (NTOU M02254) • 1 male cl 14.9 mm, 2 females cl 20.0 and 22.1 mm; stn CP2336, 09°32.4'N, 123°39.3'E, 757–729 m, 22 May 2005 (NTOU M02255) • 1 male cl 12.0 mm, 1 female cl 22.9 mm; stn CP2341,09°24.5'N, 123°49.7'E, 712–888 m, 23 May 2005 (NTOU M02256) • 1 female cl 11.1 mm; stn CP2351, 09°30.7'N, 124°3.0'E, 810–830 m, 24 May 2005 (NTOU M02257) • 1 male cl 9.0 mm; stn CP2352, 09°27.3'N, 124°3.1'E, 1260–1761 m, 24 May 2005 (NTOU M02258) • 3 males cl 11.8–20.4 mm, 5 females cl 15.7–28.0 mm; stn CP2358, 08°52.1'N, 123°37.1'E, 569–597 m, 26 May 2005 (NTOU M02259) • 1 male cl 16.2 mm; stn CP2358, 08°52.1'N, 123°37.1'E, 569–597 m, 26 May 2005 (NTOU M02260) • 6 males cl 10.4–21.9 mm, 1 ovigerous female cl 22.9 mm, 11 females cl 10.3–25.3 mm; stn CP2389, 09°27.9'N, 123°38.4'E, 784–782 m, 30 May 2005 (NTOU M02261) • 1 male cl 19.6 mm, 2 females cl 19.5 and 19.6 mm; stn CP2390, 09°27.4'N, 123°43.1'E, 627–613 m, 30 May 2005 (NTOU M02262) • 2 males cl 17.6 and 23.8 mm; stn CP2397, 09°34.9'N, 123°41.7'E, 669–712 m, 31 May 2005 (NTOU M02263) • 1 male cl 24.0 mm; stn CP2398, 09°32.6'N, 123°40.5'E, 731–741 m, 31 May 2005 (NTOU M02264) • 2 males cl 16.4 and 19.4 mm; stn CP2405, 09°39.0'N, 123°46.1'E, 387–310 m, 1 Jun. 2005 (NTOU M02265) • 1 female cl 16.6 mm; northern coast of Panglao Island, Jul. 2004–May. 2005 (NTOU M02266).

##### Diagnosis.

Rostrum bearing one pair of lateral teeth usually situated behind mid-length of rostrum. Carapace with subdorsal carinae granulate and lacking distinct spine; supraorbital and antennal spines strong; post-supraorbital spine absent; postcervical groove U-shaped in dorsal view; intermediate carina indistinct and lateral carina moderately developed. Large cheliped (pereiopod I) with inner surface of palm granular, lacking distinct spine; carpus with strong distoventral, ventro-outer (rarely absent) and dorso-inner distal spines, outer surface without distinct spine, inner surface bearing one or rarely two spines on dorsal margin; merus armed with anteroventral and subdistal dorsal spines, lacking subdistal outer spine or sharp tubercle. Pleon finely granulate, without mid-dorsal carina, pleura each with unarmed anterior margin. Telson without erected dorsal spine near base. Uropodal exopods with complete diaeresis.

##### Description.

Body covered with long or short pubescence, those on anterior two pereiopods, dorsal carapace, and pleonal tergum quite dense. Carapace finely granulated (Fig. 3A, B); rostrum 0.5–0.9× carapace length (proportionally longer in small individuals), bearing 1 pair of lateral teeth usually situated behind mid-length of rostrum, median groove extending anteriorly beyond lateral rostral teeth; subdorsal carinae granulate and lacking distinct spine; strong supraorbital and antennal spines present; post-supraorbital spine absent; cervical, postcervical, and hepatic groove well marked, with postcervical groove U-shaped in dorsal view; intermediate carina indistinct and lateral carina moderately developed; gastric tubercle near supraorbital spines, 0.3–0.4× distance between gastric tubercle and postcervical groove; distance between orbital margin and postcervical groove 1.5–1.8× distance between postcervical groove and posterior margin of carapace.

Large cheliped (pereiopod I) generally granulate (Fig. 3C–E); fingers 0.9–1.5 (mostly 1.0–1.3)× as long as palm; chela 2.6–4.1 (usually 2.8–3.2)× as long as wide and similar in both sexes, inner surface of palm granular but lacking distinct spine; carpus with strong distoventral spine, ventro-outer spine (rarely absent) and dorso-inner distal spine, outer surface without distinct spine, inner surface bearing 1 (mostly) or 2 spines on dorsal margin; merus armed with distoventral spine and subdistal dorsal spine, lacking subdistal outer spine or sharp tubercle. Pereiopod II chelate, smooth, carpus 0.5–0.7× palm length. Pereiopod III generally similar to pereiopod II but less stout; carpus 0.4–0.6× as long as palm; merus 1.6–2.2 (mostly 1.7–2.0)× as long as carpus. Pereiopods IV and V smooth, not chelate; dactyli 0.5–0.8 (mostly 0.5–0.7)× as long as propodi.

Entire pleon finely granulate (Fig. 3A, B), without mid-dorsal carina but bearing indistinct and medially interrupted transverse groove on tergites II–V and sometimes also on tergite I; pleura each with unarmed anterior margin, that of pleuron II strongly convex while those of pleura III–V only slightly convex, all terminating ventrally into sharp spine. Telson without erected dorsal spine near base.

Uropod generally smooth, exopods with distinct complete diaeresis.

Eggs spherical, 1.8–2.0 mm in diameter.

##### Color in life.

Body generally whitish to pinkish white (Fig. 5C, D), with pleon sometimes pinkish orange. Eyes whitish. Anterodorsal carapace pinkish orange. Rostrum and antennal flagella pinkish orange to orange red. Antennular flagella and maxilliped III orange red. Large cheliped whitish to pinkish orange, distal parts of fingers always pinkish orange. Pereiopods II–V whitish with distal segments orange red or entirely orange red. Pleopods whitish to orange red. Tail fan whitish, sometimes with median parts rose red. Pubescence grayish brown.

##### Etymology.

The Latin *pygmaea* (little) refers to the much smaller size of this species compared with other species in the *N. stewarti* species complex.

##### Distribution.

Western Pacific and known with certainty from southern Taiwan and the Philippines, at depths of 310–888 m, and perhaps as shallow as 170 m (see “Remarks”).

##### Remarks.

This smaller form restricted to the northwestern Pacific has a maximum carapace length of 28.0 mm (NTOU M02259), with females bearing eggs attaining only 22.9 mm in the carapace length (NTOU M02261). The largest specimens of *N. stewarti* and *N. grandis* is 54 mm (Dineshbabu 2008) and 64.1 mm (present material) in the carapace length, respectively. The smallest ovigerous females recorded for *N. stewarti* and *N. grandis* are of carapace lengths approximately 24 mm (total length 80 mm, Dineshbabu 2008) and 38 mm (body length 105 mm, Chan and Yu 1988), respectively. Other than the difference in body size, *N. pygmaea* sp. nov. is unique in the *N. stewarti* species complex in that it lacks the subdistal outer spine or sharp tubercle on the merus of the large cheliped (Fig. 3C, D), which are present in *N. stewarti* and *N. grandis* (Figs 1C, D, 2C, D).

In spite of the restricted distribution to the northwestern Pacific, *N. pygmaea* sp. nov. is genetically closer to *N. stewarti* than *N. grandis*. The lowest 16S sequence divergence between *N. pygmaea* sp. nov. and *N. stewarti* is 3.8%, whereas the sequence divergence is almost double (7.5%) between *N. pygmaea* sp. nov. and *N. grandis*. Morphologically, *N. pygmaea* sp. nov. is also generally more similar to *N. stewarti* in the surface of the pleonal tergites distinctly granular (Figs 1A, B, 3A, B), and the large cheliped is relatively less spiny (with inner surface of palm and outer surface of carpus lacking distinct spine; Figs 1C, E, 3C, E). As such a male specimen from the Philippines (NTOU M02260) has the granules arranged somewhat like a median carina on the pleon, as in some Indian *N. stewarti* specimens. Nevertheless, *N. pygmaea* sp. nov. can also be separated from *N. stewarti* by the intermediate carina on the carapace indistinct (Fig. 3A, B; vs. well-marked, Fig. 1A, B), rostral teeth usually located posterior to the mid-length of the rostrum (Fig. 3A, B; vs. usually at mid-length of the rostrum, Fig. 1A, B), and the inner surface of the carpus of the large cheliped usually armed with one or occasionally two spines along the dorsal margin (Fig. 3C; vs. usually two to four spines, rarely one spine, Fig. 1C). Of the 62 specimens examined for *N. pygmaea* sp. nov., only 10 (16.1%) have two spines instead of one on the dorsal margin of the inner surface of the carpus of the large cheliped.

The present materials from southern Taiwan and the Philippines are generally very similar. Only one specimen (NTOU M02168) has three teeth instead of one on the right side of the rostrum. As both *N. grandis* and *N. pygmaea* sp. nov. occur in the Philippines, it is necessary to re-examine the Philippines “*N. stewarti*” material reported by Macpherson (1990) to determine their exact identities. Although most of the Philippines specimens described by Macpherson (1990) are rather small, a few of them (eg. carapace length, including rostrum, 70 mm, equivalent to a carapace length of approximately 47 mm) are larger than the present largest specimen (cl 28 mm) of *N. pygmaea* sp. nov. Moreover, a Philippines specimen identified by Macpherson (1990) was obtained from a depth of 170–200 m, exceptionally shallow for species of *Nephropsis*. Reexamination of this specimen may eventually reveal that the present species or *N. grandis* extends to such shallow depth.

## Supplementary Material

XML Treatment for Nephropsis stewarti

XML Treatment for Nephropsis grandis

XML Treatment for Nephropsis pygmaea
